# Efficient bi-allelic tagging in human induced pluripotent stem cells using CRISPR

**DOI:** 10.1016/j.xpro.2023.102084

**Published:** 2023-02-01

**Authors:** Xingjie Ren, Maya Asami Takagi, Yin Shen

**Affiliations:** 1 Institute for Human Genetics, University of California, San Francisco, San Francisco, CA 94143, USA; 2 Department of Neurology, University of California, San Francisco, San Francisco, CA 94143, USA

**Keywords:** Cell Culture, Genetics, Molecular Biology, CRISPR, Stem Cells

## Abstract

Allelic tagging of endogenous genes enables studying gene function and transcriptional control in the native genomic context. Here, we present an efficient protocol for bi-allelic tagging of protein-coding genes with fluorescent reporters in human iPSCs using the CRISPR-Cas9-mediated homology-directed repair. We detail steps for design, cloning, electroporation, and single-cell clone isolation and validation. The tagging strategy described in this protocol is readily applicable for knockin of other reporters in diverse cell types for biomedical research.

## Highlights

• Genome editing in human iPSCs using CRISPR-Cas9 ribonucleoprotein
• Tagging genes on both alleles with distinct reporters in iPSCs
• Deriving clonal iPSC lines leveraging single-cell FACS

## Abstract (editor highlights)

Publisher’s note: Undertaking any experimental protocol requires adherence to local institutional guidelines for laboratory safety and ethics.

## Graphical abstract

**Figure:**
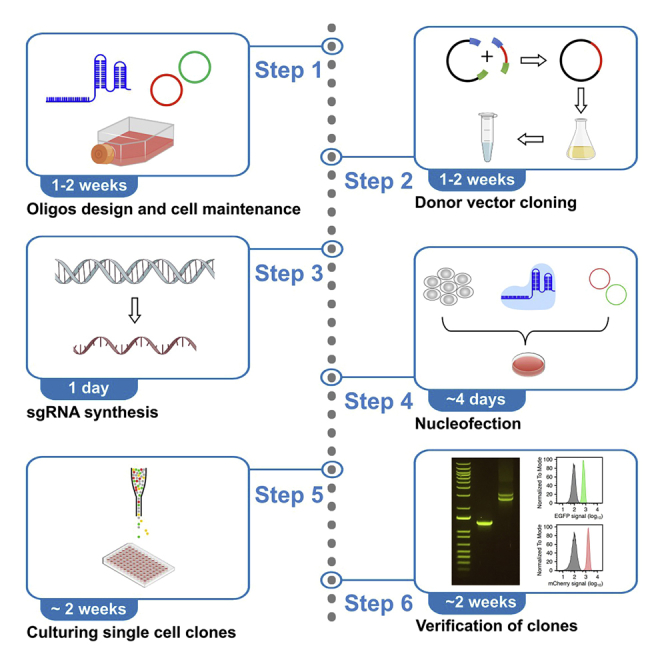

## Before you begin

### Preparation one: Design and order DNA oligos

**Timing: 1 h (excluding the time of ordering and delivery of DNA oligos)**

Here, we describe the design of gene-specific sgRNA and donor vector for tagging gene of interest with CRISPR-Cas9 mediated homology directed repair.^1^^,^^2^ The sgRNA will be synthesized via *in vitro* transcription, and the donor vectors will be constructed using Gibson assembly.

1. Design the sgRNA.
  a. Find the genomic coordinate of the region centered on the knock-in site.
    ***Note:*** The protein tags can be inserted into the N-terminal or C-terminal of the protein of your interest for specific purposes. We inserted EGFP and mCherry tags into C-terminal of SIN3A, and selected the 50 bp upstream and downstream of stop codon of *SIN3A* as target site for sgRNA design.
  b. Design the sgRNA using online CRISPR sgRNA design program, such as CHOPCHOP (https://chopchop.cbu.uib.no)^3^ or GuideScan (https://guidescan.com).^4^
    ***Note:*** Select sgRNA based on the following two criteria: (1) specificity with least predicted off-target effects (without perfectly matched or one to two mismatched off-target sites) and (2) located within 50 bp upstream or downstream of knock-in site. As shown in Figure 1A, we picked two sgRNAs for gene *SIN3A*.
  c. Design the DNA oligos for *in vitro* sgRNA transcription using the template in Figure 1B.
    ***Note:*** The sgRNA search window can be increased to 100 bp upstream or downstream of knock-in site of gene of interest if no sgRNA available from the design. Please note that the knock-in efficiency is decreased rapidly with increasing distance between knock-in site and sgRNA cut site.^5^ Two sgRNAs can increase the knock-in efficiency, however, the second sgRNA is not necessary if only one high-quality sgRNA can be designed.
    **Figure 1:**
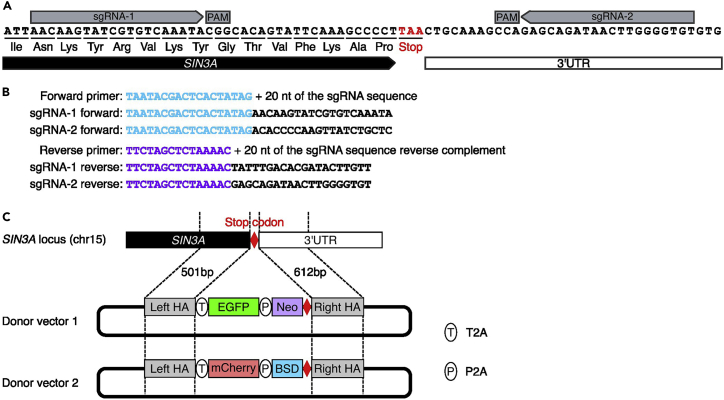
Schematic of the sgRNA design for *SIN3A* bi-allelic tagging (A) Two sgRNAs were designed for *SIN3A* bi-allelic tagging. sgRNA-1 is located in upstream of the *SIN3A* stop codon, and sgRNA-2 is located in downstream of the *SIN3A* stop codon. (B) The oligos used for *in vitro* transcription of sgRNA-1 and sgRNA-2. The T7 promoter in the forward primers is labeled skyblue, and the partial sgRNA scaffold sequence in the reverse primers is labeled purple. (C) Schematic of the EGFP and mCherry donor design for *SIN3A* bi-allelic tagging. The sequences upstream and downstream of the *SIN3A* stop codon were selected as the left and right homology arms for C-terminal bi-allelic tagging.

2. Design the donor vector.
  a. Design the left and right homology arms.
    ***Note:*** The homology arms are approximately 500–1,000 bp upstream and downstream of the knock-in site. See Figure 1C for a schematic of donor vectors used for *SIN3A* as an example. To pick the genomic region as homology arm, we designed two pairs of primers 500–1,000 bp upstream of *SIN3A* stop codon and 500–1,000 bp downstream of *SIN3A* stop codon, respectively, by using Primer-BLAST (https://www.ncbi.nlm.nih.gov/tools/primer-blast/). The primers can also be designed by using other programs. The regions between the forward and reverse primers are used as homology arms. We chose a 501 bp sequence upstream of *SIN3A* stop codon as the left homology arm (SIN3A left in Figures 2A and 2B), and a 612 bp sequence downstream of *SIN3A* stop codon as the right homology arm (SIN3A right in Figures 2A and 2B).
    **Figure 2:**
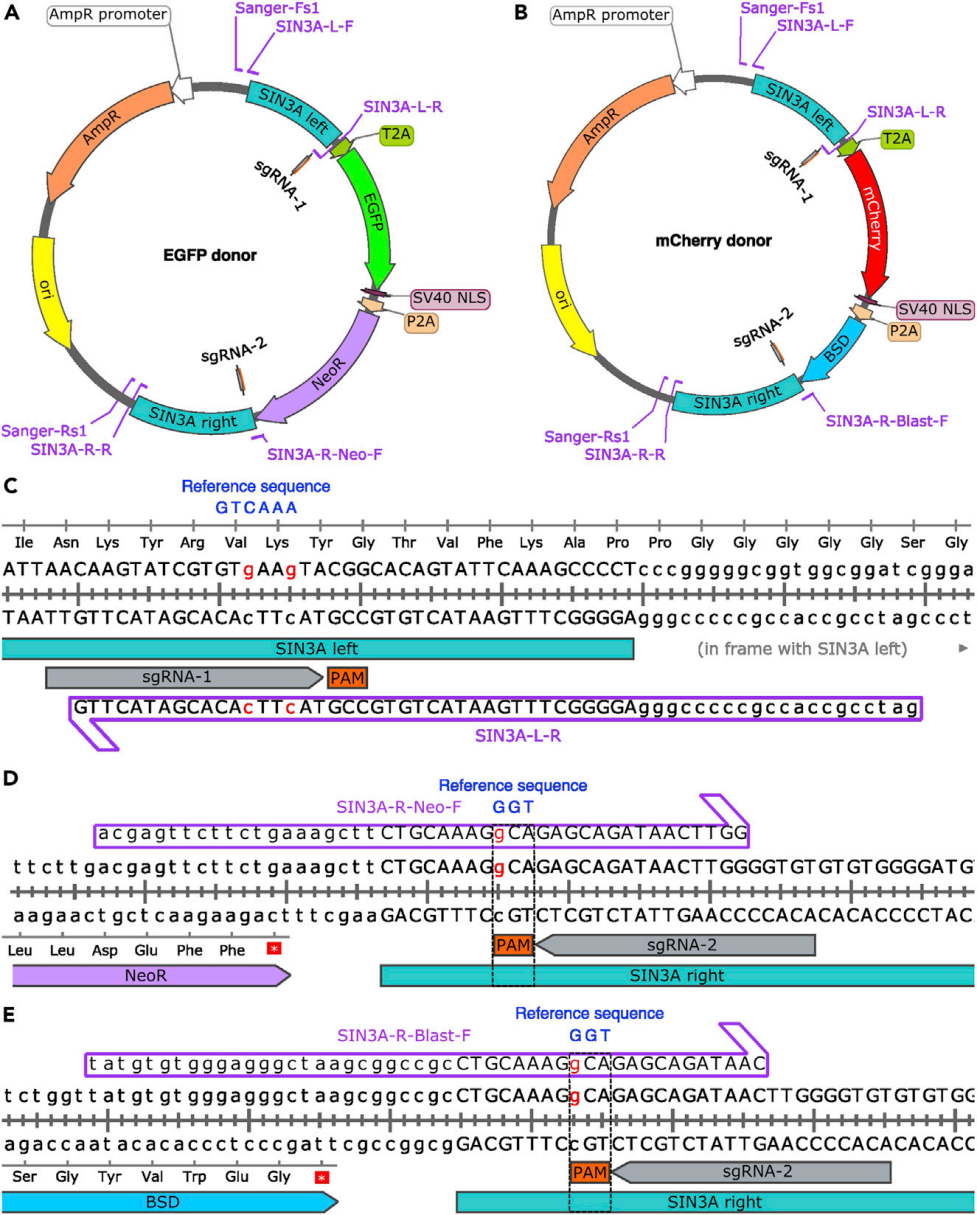
Design of EGFP and mCherry donor vectors (A and B) The EGFP and mCherry donor vectors for *SIN3A* bi-allelic tagging. The left homology arm is the sequence preceding the *SIN3A* stop codon, and the right homology arm is the sequence following the *SIN3A* stop codon. EGFP, mCherry, neomycin, and blasticidin sequences are used for the bi-allelic tagging of *SIN3A*. T2A and P2A self-cleaving peptides are used for the translation of multiple proteins from the same mRNA transcript. (C) Example of a primer for left homology arm insertion. Silent mutations are made in the sgRNA spacer to inhibit the cutting of donor vectors by the CRISPR-Cas9 system. (D and E) Examples of primers for right homology arm insertion. A point mutation is made in the PAM sequence to disrupt the cutting of donor vectors by the CRISPR-Cas9 system.
  b. Design the DNA oligos for Gibson assembly mediated donor vector cloning.
    ***Note:*** Each oligo should contain a partial sequence from donor vector and partial sequence from homology arm of the target gene. Figure 2 and Table 1 illustrate the oligo design for the donor vector used for *SIN3A* bi-allelic tagging. To prevent the cutting of donor vectors from CRISPR-Cas9 system, we introduced point mutations in the sgRNA spacer or PAM sequence in DNA oligos (Figures 2C–2E).
    **Table 1:** Oligos for amplifying the homology arms and Sanger sequencing

Oligo name	Sequence
SIN3A-L-F	tactgagagtgcaccccgggGAACAGCGAGCTTTTTCAGCC
SIN3A-L-R	gatccgccaccgcccccgggAGGGGCTTTGAATACTGTGCCGTACTTCACACGATACTTG
SIN3A-R-Neo-F	acgagttcttctgaaagcttCTGCAAAGGCAGAGCAGATAACTTGG
SIN3A-R-Blast-F	tatgtgtgggagggctaagcggccgcCTGCAAAGGCAGAGCAGATAAC
SIN3A-R-R	ccatgattacgccgcggccgcAGGCAGCAAGGGAATCGTC
Sanger-Fs1	agcgggtgttggcgggtgtcgg
Sanger-Rs1	ccggctcgtatgttgtgtgg
  c. Design the primers for Sanger sequencing to check the sequences of homology arms in donor vectors.
    ***Note:*** The location of Sanger sequencing primers is about 50 bp away from the insert. See Figure 2 and Table 1 for the examples of the Sanger sequencing primers used for SIN3A donor vectors.
    ***Note:*** For the bi-allelic tagging purpose, two donor vectors have different tags but same homology arms. For example, we designed two donor vectors with the same homology arms, but one contains EGFP and another contains mCherry for *SIN3A* bi-allelic tagging. Several studies have demonstrated that incorporating blocking mutation in sgRNA PAM or spacer sequence in donor template can increase knock-in efficiency.^5^^,^^6^^,^^7^ Consider using synonymous mutations when introducing mutations at sgRNA spacer or PAM sequences in the homology arms to prevent editing to the donor vector. The knock-in efficiency positively correlates with the length of homology arms.^8^^,^^9^ Larger vectors lead to decreased transfection efficiency and cell viability.^10^
    ***Note:*** Basic cloning vector can be used as backbone for donor vector, for example pUC19. Here, we used the vectors containing fluorescent proteins as backbone for SIN3A donor vectors cloning. For each DNA oligo, the capital letters indicate the sequences from homology arms, the lowercase letters indicate the sequences from donor vector backbone, and the underlined capital letters indicate the introduced mutations for preventing the cutting of donor vector from CRISPR-Cas9 system. Please check Figures 2A and 2B for the position of each oligo.

3. Order DNA oligos for *in vitro* sgRNA transcription and Gibson assembly as 25 nmol DNA oligos with standard desalting from IDT or other companies. In addition, order Cas9-NLS protein from NEB, Synthego, or other alternative vendor. You can also order Cas9-NLS protein from QB3 MacroLab in University of California, Berkeley.

### Preparation two: Human induced pluripotent stem cells maintenance

**Timing: 1–2 weeks**

Here, we describe the procedure for thawing, passaging, and cryopreserving human induced pluripotent stem cell (iPSC) lines. The information is provided for optimizing iPSCs growth, nucleofection, and isolating single clones with desired genotyping. We use WTC11-i^3^N iPSC line^11^ as an example for bi-allelic tagging of *SIN3A*.

4. Cell culture plate preparation with Matrigel coating.
  a. Thaw Corning Matrigel hESC-qualified Matrix on ice in a 4°C refrigerator for >12 h.
  b. Once the Matrigel is thawed, swirl the vial to ensure that Matrigel is homogeneous.
  c. Dispense each aliquot with the amount making 25 mL Matrigel after dilution in pre-cooled 50 mL conical tubes.
  d. Store the aliquot in −80°C.
  e. Thaw one frozen aliquot of the Matrigel on ice for at least 2 h. Dilute the aliquot with 25 mL of cold DMEM/F-12 medium, and processed to the next step or store at 4°C for up to 1 week.
  f. Coat a cell culture plate with prepared Matrigel by adding an appropriate amount of Matrigel into the plate (Table 2). Incubate the plate with Matrigel in a 37°C / 5% CO_2_ incubator for at least 30 min before use.

**Table 2:** The amount of reagents used for iPSC maintenance and passaging

	Matrigel (mL/well)	Medium (mL/well)	Accutase (mL/well)
6-well plate	1 mL	2 mL	0.5 mL
24-well plate	0.25 mL	0.5 mL	0.15 mL
96-well plate	0.05 mL	0.15–0.2 mL	N/A

***Optional:*** The Matrigel can be collected and reused if the incubation time is less than 2 h.

5. Thawing iPSCs.
  a. Prepare Matrigel coated plate as described in steps 4e-4f.
  b. Label the plate with cell line, passage number, date, and operator initials.
  c. Remove vial of frozen iPSC from the liquid nitrogen tank and place it on dry ice.
  d. Add 10 mL DMEM/F-12 medium into a 15 mL conical tube.
  e. Thaw vial in a 37°C water bath until only a small ice pellet can be seen. Quickly transfer the cell suspension to the prepared 15 mL conical tube using P1000 and mix 2–3 times.
  f. Centrifuge the conical tube for 5 min in a swinging-bucket centrifuge at 200 × *g* and 20°C–25°C.
  g. Carefully aspirate the supernatant and resuspend the cell pellet in an appropriate volume (see Table 2 for volume) mTeSR1 medium with Rock inhibitor (final concentration is 10 μM).
  h. Remove the Matrigel from coated well and add cell suspension to the well.
  i. Place in a 37°C / 5% CO_2_ incubator, gently shaking plate left/right and up/down to ensure even seeding.
  j. Replace media daily with mTeSR1 (without Rock inhibitor). Once until cells reach 80%–90% confluency, they need to be passaged.
6. Passaging iPSCs.
  a. Prepare a Matrigel coated plate as described in steps 4e-4f.
  b. Label the plate with cell line, passage number, date, and operator initials.
  c. Remove 80%–90% confluent plate from incubator and aspirate spent media. Wash the plate with DPBS, using a volume equal to the volume of culture medium.
  d. Aspirate DPBS and add Accutase (see Table 2 for volume).
  e. Incubate the plate at 37°C for 5 min. Check cells for detachment by gently shake the plate. If < 90% detachment is observed, incubate the plate for an additional 1–2 min, and check again until 90% detachment is observed.
  f. Add DMEM/F12 (2× of the volume of Accutase) and mix the cell suspension up and down. Transfer the cell suspension to an appropriately sized conical tube. Wash the plate with DMEM/F12 again (2× the volume of Accutase) and transfer into the same conical tube.
  g. Centrifuge at 200 × *g* for 5 min at 20°C–25°C.
  h. Aspirate supernatant and resuspend cell pellet in an appropriate volume of mTeSR1 with Rock inhibitor (see Table 2 for volume).
  i. Aspirate the Matrigel from coated well and seed cells into the well with splitting ratio of 1:6 to 1:10.
  j. Place in a 37°C / 5% CO_2_ incubator, gently shaking plate left/right and up/down to ensure even seeding.
  k. Replace media daily with mTeSR1 (without Rock inhibitor) until cells reach 80%–90% confluence.
7. Freezing iPSCs.
  a. Prepare cryovials by labeling with cell line, passage number, number of cells per vial, date, and operator initials.
  b. Collect the cells from the plate as cell pellet using the same procedure in passaging step.
  c. Aspirate supernatant and resuspend cell pellet in an appropriate volume of freezing medium.
  d. Dispense cell suspension into pre-labeled cryovials and place vials in a freezing container.
  e. Freeze the container at −80°C for 24 h then transfer the vials to liquid nitrogen for long-term storage.

## Key resources table

**Table:** 

REAGENT or RESOURCE	SOURCE	IDENTIFIER
**Bacterial and virus strains**		

Stellar Chemically Competent Cells	Takara	Cat# 636763

**Chemicals, peptides, and recombinant proteins**		

Matrigel	Corning	Cat# 354277
mTeSR1	STEMCELL Technologies	Cat# 85850
DMEM/F-12	Gibco	Cat# 11330032
Y-27632 (Rock inhibitor)	STEMCELL Technologies	Cat# 72304
DPBS	Life Technologies	Cat# 14190-144
LB broth	Thermo Fisher Scientific	Cat# BP9722-500
LB agar	Thermo Fisher Scientific	Cat# BP9745-500
QuickExtract DNA Extraction Solution	Lucigen	Cat# QE09050
Ampicillin sodium salt	Thermo Fisher Scientific	Cat# BP1760-25
Penicillin-Streptomycin	Gibco	Cat# 15070063
EDTA	Invitrogen	Cat# 15575020
HEPES	Gibco	Cat# 15630080
Fetal bovine serum	HyClone	Cat# SH30396.03
CloneR	STEMCELL Technologies	Cat# 05888
CloneR 2	STEMCELL Technologies	Cat# 100-0691
Essential 8 Medium	Thermo Fisher Scientific	Cat# A1517001
XmaI	NEB	Cat# R0180S
NotI	NEB	Cat# R0189S
Accutase	STEMCELL Technologies	Cat# 07920
TE buffer	Thermo Fisher Scientific	Cat# 12090015
Nuclease-free water	Thermo Fisher Scientific	Cat# R0582

**Critical commercial assays**		

Human Stem Cell Nucleofector Kit 1	Lonza	Cat# VPH-5012
E.Z.N.A. Plasmid DNA Mini Kit	Omega Bio-tek	Cat# D6942-02
Wizard SV Gel and PCR Clean-Up System	Promega	Cat# A9282
QIAGEN Plasmid Plus Midi Kit	QIAGEN	Cat# 12943
Wizard SV Genomic DNA Purification System	Promega	Cat# A2360
NEBNext High-Fidelity 2× PCR Master Mix	NEB	Cat# M0541S
NEBuilder HiFi DNA Assembly Master Mix	NEB	Cat# E2621S
GoTaq Green Master Mix	Promega	Cat# M7123
Precision sgRNA Synthesis Kit	Invitrogen	Cat# A29377
QIAGEN RNeasy Plus Mini Kit	QIAGEN	Cat# 74134
iScript cDNA synthesis kit	Bio-Rad	Cat# 1708891
Qubit dsDNA HS Assay Kit	Thermo Fisher Scientific	Cat# Q32851
0.22 μm filtration system	Millipore	Cat# SCGP00525

**Experimental models: Cell lines**		

WTC11-i^3^N	From Gan Lab	PMID: 28966121

**Oligonucleotides**		

sgRNA-1-forward: TAATACGACTCACTATAGAACAAGTATCGTGTCAAATA	IDT	N/A
sgRNA-1-reverse: TTCTAGCTCTAAAACTATTTGACACGATACTTGTT	IDT	N/A
sgRNA-2-forward: TAATACGACTCACTATAGACACCCCAAGTTATCTGCTC	IDT	N/A
sgRNA-2-reverse: TTCTAGCTCTAAAACGAGCAGATAACTTGGGGTGT	IDT	N/A
SIN3A-L-F: tactgagagtgcaccccgggGAACAGCGAGCTTTTTCAGCC	IDT	N/A
SIN3A-L-R: gatccgccaccgcccccggg AGGGGCTTTGAATACTGTGCCGTACTTCACACGATACTTG	IDT	N/A
SIN3A-R-Neo-F: acgagttcttctgaaagcttCTGCAAAGGCAGAGCAGATAACTTGG	IDT	N/A
SIN3A-R-Blast-F: tatgtgtgggagggctaagcggccgcCTGCAAAGGCAGAGCAGATAAC	IDT	N/A
SIN3A-R-R: ccatgattacgccgcggccgcAGGCAGCAAGGGAATCGTC	IDT	N/A
Sanger-Fs1: agcgggtgttggcgggtgtcgg	IDT	N/A
Sanger-Rs1: ccggctcgtatgttgtgtgg	IDT	N/A
gF1: TCCCTCGGTTATACTAACGCATG	IDT	N/A
gR1: AGCTGGTGGATGGCACTAAG	IDT	N/A
EGFP-R: CTTCATGTGGTCGGGGTAGC	IDT	N/A
mCherry-R: CAAGTAGTCGGGGATGTCGG	IDT	N/A
Neo-F: CGATGATCTCGTCGTGACCC	IDT	N/A
BSD-F: GTACCGCCACCATGGCCAAGCCTTTGTCTCA	IDT	N/A
NANOG-F: GTCCCAAAGGCAAACAACCC	IDT	N/A
NANOG-R: GCTGGGTGGAAGAGAACACA	IDT	N/A
POU5F1-F: TGCAGGCCCGAAAGAGAAAG	IDT	N/A
POU5F1-R: GATCTGCTGCAGTGTGGGTT	IDT	N/A
SOX2-F: TACAGCATGATGCAGGACCAG	IDT	N/A
SOX2-R: AGCCGTTCATGTAGGTCTGC	IDT	N/A

**Recombinant DNA**		

SIN3A EGFP donor	This paper	N/A
SIN3A mCherry donor	This paper	N/A

**Software and algorithms**		

SnapGene	SnapGene	https://www.snapgene.com/

**Other**		

6-well plate	GenClone	Cat# 25-105
24-well plate	GenClone	Cat# 25-107
96-well plate	GenClone	Cat# 25-109
PCR 8-tube stripes	USA Scientific	Cat# 1402-4700
Nucleofector 2b Device	Lonza	Cat# AAB-1001
FACS tubes	Falcon	Cat# 352235
15 mL polystyrene conical tube	Corning	Cat# 352096
Freezing tubes	Greiner Bio-One	Cat# 122277
NanoDrop	Thermo Fisher Scientific	N/A
Qubit 2.0 Fluorometer	Invitrogen	Cat# Q32866

## Materials and equipment

**Table:** DNA oligo solution

Reagent	Amount	Final stock concentration	Final working stock concentration
DNA oligo	Whole oligo	100 μM	20 μM
TE buffer	Variable	N/A	N/A

***Note:*** Store the resuspended DNA oligos at −20°C for up to years.

**Table:** Matrigel solution

Reagent	Final concentration	Amount
Matrigel	100 μg/mL	Variable
DMEM/F-12	N/A	25 mL

***Note:*** Store the Matrigel solution at 4°C for up to 1 week. The amount of Matrigel is calculated based on the protein concentration of Matrigel stock.

**Table:** iPSC culture medium

Reagent	Final concentration	Amount
mTeSR1 basal medium	N/A	400 mL
mTeSR1 5× supplement	1×	100 mL
Total	N/A	500 mL

***Note:*** Store the medium at 4°C for up to 2 weeks and −20°C for up to 6 months.

**Table:** FACS buffer

Reagent	Final concentration	Amount
0.5 M EDTA	2 mM	0.2 mL
1 M HEPES pH 7.0	25 mM	1.25 mL
Fetal Bovine Serum	1%	0.5 mL
Penicillin-Streptomycin	1%	0.5 mL
DPBS	N/A	47.55 mL
Total	N/A	50 mL

***Note:*** The FACS buffer should be filtered with 0.22 μm filtration system and stored at 4°C for up to 2 months.

## Step-by-step method details

### Cloning donor vectors

**Timing: 1–2 weeks (for steps 1 to 17)**

The purpose of this step is to clone the donor vectors for tagging the target gene. The left and right homology arms are inserted into the donor using Gibson assembly.

1. Isolate genomic DNA from the desired cell line for tagging using Wizard SV Genomic DNA Purification System following the manufacturer’s protocol (https://www.promega.com/-/media/files/resources/protcards/wizard-genomic-dna-purification-kit-quick-protocol.pdf?rev=4cc2e14ff84c4281a97eb50b32755c33&sc_lang=en).

***Note:*** The isolated genomic DNA will be used as template for amplification of homology arms. We isolated genomic DNA from WTC11-i^3^N iPSCs,^11^ which is the parental line we used for *SIN3A* bi-allelic tagging.

2. Amplify the left and right homology arms from the isolated genomic DNA using NEBNext High-Fidelity 2× PCR Master Mix and DNA oligos listed in Table 1. Set up two PCR reactions for each homology arm as below.

**Table:** 

Component	Amount (50 μL reaction)
20 μM forward primer	0.5 μL
20 μM reverse primer	0.5 μL
Genomic DNA (200 ng)	Variable
NEBNext High-Fidelity 2× PCR Master Mix	25 μL
Nuclease-free water	To 50 μL

***Note:*** The homology arms can be amplified using other high fidelity DNA polymerase.

3. Perform amplification using the cycling parameters below.

**Table:** 

Steps	Temperature	Time	Cycles
Initial Denaturation	98°C	1 min	1
Denaturation	98°C	15 s	30
Annealing	55°C	30 s	
Extension	72°C	1 min	
Final extension	72°C	1 min	1
Hold	4°C	Forever

***Note:*** The temperature used for amplification depends on DNA polymerase and DNA oligos.

**Figure 3:**
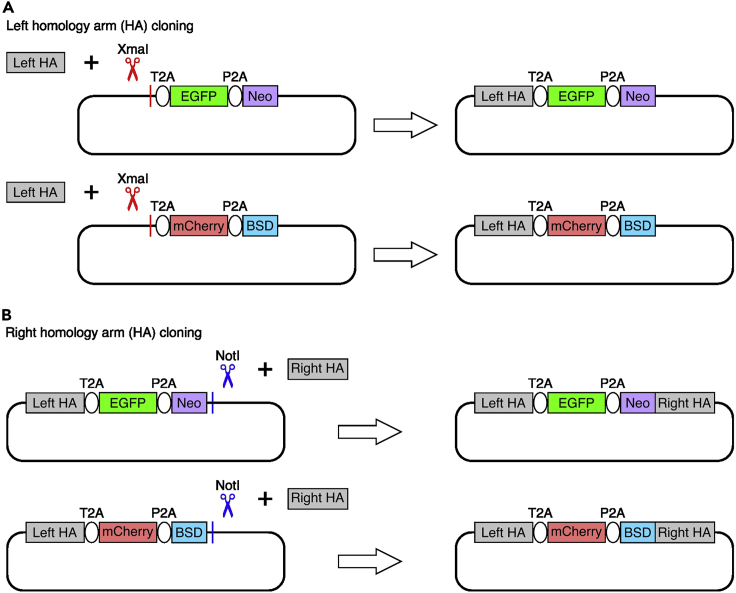
The cloning strategy for SIN3A donor vectors (A) Insertion of the left homology arm by Xmal linearization of backbone followed by left homology arm insertion with Gibson assembly. (B) Insertion of the right homology arm by NotI linearization of the vectors from step A followed by right homology arm insertion with Gibson assembly.

4. Digest the backbone (the EGFP donor and mCherry empty donor vectors without homology arm) using the reaction below for left homology arm insertion (Figure 3A). Incubate the digestion reaction at 37°C for 1 h.

**Table:** 

Component	Amount (50 μL reaction)
EGFP / mCherry empty donor	2,000 ng
XmaI	1 μL
rCutSmart buffer (10×)	5 μL
Nuclease-free water	To 50 μL

***Note:*** The backbones we used are two premade pUC57 based empty donor vectors in our lab, which contain either EGFP or mCherry fluorescent protein without homology arms. Other basic cloning vectors can also be used as backbone for donor vector cloning, for example pUC19. The reporters used for tagging can be inserted into the basic cloning vector before homology arms cloning, or together with homology arms using multiple fragment assembly.

5. Purify the digested backbone and PCR amplified left and right homology arms using gel extraction and Wizard SV Gel and PCR Clean-Up System. Follow the manufacturer’s protocol and elute in 50 μL elution buffer (https://www.promega.com/-/media/files/resources/protcards/wizard-sv-gel-and-pcr-clean-up-system-quick-protocol.pdf?rev=29aa4610719c4f96afd213c345bc76d3&sc_lang=en). Check the concentration of each product via NanoDrop.
6. Set up one Gibson assembly reaction as below for EGFP donor, and one for mCherry donor separately. Incubate the reaction at 50°C for 1 h to insert the left homology arm into the digested EGFP and mCherry empty donor vectors.

**Table:** 

Component	Amount
EGFP / mCherry empty donor fragment	50 ng
Left homology arm fragment	2–5 fold molar ratio to donor fragment
NEBuilder HiFi DNA Assembly Master Mix	10 μL
Nuclease-free water	To 20 μL

7. Transform Stellar chemically competent cells with 2 μL of the chilled assembled product, following the transformation protocol (https://www.takarabio.com/documents/User Manual/PT5055/PT5055-2.pdf).
8. Perform 8 or 16 colony PCR reactions for each donor vector and set up the colony PCR reactions as below. The use of one primer targeting backbone and one primer targeting insert can reduce the false positive colonies.

**Table:** 

Component	Amount (20 μL reaction)
20 μM forward primer	0.5 μL
20 μM reverse primer	0.5 μL
GoTaq Green Master Mix	10 μL
Nuclease-free water	9 μL

***Note:*** We used Sanger-Fs1 and SIN3A-L-R for left homology arm confirmation, SIN3A-R-Neo-F, SIN3A-R-Blast-F and Sanger-Rs1 for right homology arm confirmation (see Table 1 for oligo sequences).

9. Perform amplification using the cycling parameters below. Run the PCR product on the agarose gel and select the colonies with successful insertion based on the size of PCR product.

**Table:** 

Steps	Temperature	Time	Cycles
Initial Denaturation	95°C	2 min	1
Denaturation	95°C	30 s	32
Annealing	55°C	30 s	
Extension	72°C	1 min	
Final extension	72°C	5 min	1
Hold	4°C	Forever

10. Culture 3–5 positive colonies using 6 mL LB broth with Ampicillin for each and extract plasmid DNA by E.Z.N.A. Plasmid DNA Mini Kit using the manufacturer’s protocol (https://ensur.omegabio.com/ensur/contentAction.aspx?key=Production.3647.S2R4E1A3.20190204.67.4680522).
11. Confirm the sequence of left homology arm by Sanger sequencing using the Sanger sequencing primer Sanger-Fs1 in Table1.
12. Analyze the Sanger sequencing results and confirm the sequence of left homology arm in both EGFP and mCherry donors with Snapgene.
13. Pick one EGFP donor and one mCherry donor with the correct left homology arm sequence for right homology arm insertion.
14. Prepare the digestion reaction for the EGFP and mCherry donor vectors with left homology arm with NotI (Figure 3B). Incubate the reaction at 37°C for 1 h.

**Table:** 

Component	Amount (50 μL reaction)
EGFP / mCherry donor vector with left homology arm	2,000 ng
NotI	1 μL
NEBuffer r3.1 (10×)	5 μL
Nuclease-free water	To 50 μL

15. Purify the digested vectors from step 14 using gel extraction and Wizard SV Gel and PCR Clean-Up System. Follow the manufacturer’s protocol and elute in 50 μL elution buffer (https://www.promega.com/-/media/files/resources/protcards/wizard-sv-gel-and-pcr-clean-up-system-quick-protocol.pdf?rev=29aa4610719c4f96afd213c345bc76d3&sc_lang=en). Check the concentration of each product with NanoDrop.
16. Repeat steps 6–11 to insert the right homology arm into donor vectors, and check the sequence of right homology arm using the Sanger sequencing primer Sanger-Rs1 in Table 1.
17. Make midi-prep for EGFP and mCherry donor vectors with both left and right homology arms using QIAGEN Plasmid Plus Midi Kit following manufacturer’s protocol (https://www.qiagen.com/us/Resources/ResourceDetail?id=3da21fc3-a078-4665-aefe-06154db2b6d2&lang=en).

***Note:*** We will add the donor plasmids together with Cas9/sgRNA ribonuclease protein complex into nucleofection reaction in later step. Their volume should not exceed 10% of the total reaction volume. We usually elute the donor vectors from midi-prep with 100 μL elution buffer to make the final concentration of each donor vector around 2,000 ng/μL. The quality of donor plasmid is important for successful tagging. We checked the integrity of donor vectors before use by using agarose gel electrophoresis.

### Synthesis of sgRNA

**Timing: 1 day (for steps 18 to 20)**

The purpose of this step is to synthesize sgRNA using the precision sgRNA synthesis kit, which is a complete *in vitro* transcription system for rapid synthesis and purification of sgRNA ready for nucleofection.

18. PCR assemble the sgRNA DNA template.
  a. Set up the PCR assembly reaction as below to amplify the sgRNA DNA template. The DNA oligos are listed in Figure 1B.

**Table:** 

Component	Amount
Phusion High-Fidelity PCR Master Mix (2×)	12.5 μL
Tracr Fragment + T7 Primer Mix	1 μL
0.3 μM sgRNA forward and reverse oligo mix	1 μL
Nuclease-free water	10.5 μL
  b. Perform assembly PCR using the cycling parameters below.

**Table:** 

Steps	Temperature	Time	Cycles
Initial Denaturation	98°C	10 s	1
Denaturation	98°C	5 s	32
Annealing	55°C	15 s	
Final extension	72°C	1 min	1
Hold	4°C	Forever
  c. Confirm the template assembly by running 5 μL of the PCR product against a DNA ladder on a 2% Agarose Gel. The correct size of sgRNA DNA template is about 120 bp.
19. Perform *in vitro* transcription.
  a. Set up the following *in vitro* transcription (IVT) reaction, adding the reaction components in the order given.

**Table:** 

Component	Amount
NTP mix (100 mM each of ATP, GTP, CTP, UTP)	8 μL
sgRNA DNA template (from step 18)	6 μL
5× TranscriptAid Reaction Buffer	4 μL
TranscriptAid Enzyme Mix	2 μL
  b. Mix the reaction components thoroughly, centrifuge briefly to collect all drops, and incubate at 37°C for 2 h.
    ***Note:*** You can set up multiple transcription reactions or extend the incubation up to 4 h for higher sgRNA yields. In our hand, each transcription reaction can produce about 40 μg sgRNA at 2 h of incubation.
  c. Add 1 μL of DNase I into the reaction mix after the transcription reaction, mix by pipetting, and incubate at 37°C for 15 min to remove the sgRNA DNA template.
  d. Dilute 0.5 μL of the IVT product in 10 μL of nuclease-free water, and mix with RNA loading dye.
  e. Heat the sample at 70°C for 10 min to denature the sgRNA and chill on ice, then run on a 2% Agarose Gel against an RNA Ladder to check the integrity of synthesized sgRNA. The expected sgRNA transcript size is 100 bases.
20. Purify *in vitro* transcribed sgRNA.
  a. Adjust the volume of the IVT product to 200 μL with nuclease-free water.
  b. Add 100 μL of Binding Buffer. Mix thoroughly by pipetting.
  c. Add 300 μL of ethanol (>96%) and mix by pipetting.
  d. Transfer the mixture to the GeneJET RNA Purification Micro Column and centrifuge for 30–60 s at 14,000 × *g*. Discard the flow-through.
  e. Add 700 μL Wash Buffer 1 and centrifuge for 30–60 s at 14,000 × *g*. Discard the flow-through.
  f. Add 700 μL Wash Buffer 2 and centrifuge for 30–60 s at 14,000 × *g*. Discard the flow-through and repeat.
  g. Centrifuge the empty purification column for an additional 60 s at 14,000 × *g* to completely remove any residual Wash Buffer and transfer the purification column to a clean 1.5 mL collection tube.
  h. Add 15 μL of nuclease-free water to the center of the purification column filter, and centrifuge for 60 s at 14,000 × *g* to elute the sgRNA.
  i. Check the concentration of the sgRNA with NanoDrop or Qubit. Make 4 μg aliquots for each sgRNA with PCR tubes and freeze them at −80°C. sgRNA is generally stable at −80°C for more than one year without degradation.

### Deliver CRISPR-Cas9 genome editing reagents into cells using nucleofection

**Timing: 3–4 days (for steps 21 to 36)**

The purpose of this step is to deliver the *in vitro* assembled Cas9/sgRNA ribonucleoprotein complex and donor vectors into the cells using nucleofection. We used the Lonza Nucleofector 2b Device and Human Stem Cell Nucleofector Kit 1 for nucleofection of iPSCs.

21. Prepare the iPSCs for nucleofection. Grow 1 million iPSCs for 1 nucleofection.

***Note:*** High quality iPSCs with normal morphology and growth rate are very important for successful tagging. The iPSCs are ready for nucleofection when they reach to 80%–90% confluency. Meanwhile, maintain the growth of iPSC, which will be used as control in the cell sorting step.

22. Prepare the Matrigel coated well for seeding nucleofection cells.

***Note:*** We usually prepare 2 wells of a 6-well plate for 1 nucleofection.

23. Assemble the Cas9/sgRNA ribonuclease protein complex *in vitro* (Tube 1) and prepare donor plasmids (Tube 2) as below. Incubate them at 20°C–25°C for 15–20 min.

**Table:** 

Tube 1: Cas9/sgRNA complex		Tube 2: Donor plasmids	
Cas9-NLS protein	16 μg	EGFP donor	3 μg
sgRNA 1	4 μg	mCherry donor	3 μg
sgRNA 2	4 μg

***Note:*** Make sure the total volume of tube 1 and tube 2 does not exceed 10 μL.

24. Harvest the cells by following steps 6c–6f in before you begin part.
  a. Count and aliquot 1 million cells into one 15 mL conical tube.
  b. Centrifuge the 1 million cells at 200 × *g* for 5 min at 20°C–25°C.
25. During centrifuging, prepare 4 mL mTeSR1 medium with Rock inhibitor for 1 nucleofection.
26. Remove the Matrigel from the 6-well plate and add 1 mL of the prepared medium into each well.
27. Transfer the conical tube with cell pellet back to cell culture hood when centrifuging is done and proceed to the next step.
28. Combine 82 μL nucleofector solution with 18 μL supplement from Lonza Human Stem Cell Nucleofector Kit 1, and then combine with Cas9/sgRNA complex and donor plasmids together.
29. Remove the supernatant completely from the cell pellet. Resuspend the cell pellet carefully with the mixed nucleofection reagents from step 28.
30. Transfer the cell suspension into the nucleofector cuvette using a P200 pipet and avoid creating any bubbles.

***Note:*** Any bubbles in the cuvette will destroy the nucleofection reaction or decrease the transfection efficiency.

31. Gently tap the nucleofector cuvette to make sure the sample covers the bottom of the cuvette.
32. Transfer the cuvette with a closed lid to Lonza nucleofector and start nucleofection process.

***Note:*** We used Lonza nucleofector 2b device with program A-023.

33. Take out the cuvette immediately when nucleofection process is finished.
34. Resuspend the cells in the cuvette with the prepared mTeSR1 medium.
  a. Add 1 mL of the prepared mTeSR1 medium from step 25 into the cuvette.
  b. Transfer the cell suspension to the prepared Matrigel coated wells from step 26 using the provided single use pipet by adding 0.5 mL into each well.
  c. Wash the cuvette with 1 mL medium again and transfer 0.5 mL to each well.

***Note:*** In this step, we seed the 1 million cells after nucleofection into 2 wells of a 6-well plate with 0.5 million per well.

35. Gently shake the plate left/right and forward/backward to ensure even seeding. Incubate the cells in a 37°C / 5% CO_2_ incubator.
36. 24 h after nucleofection, change the culture medium daily with mTeSR1 medium without Rock inhibitor and grow the nucleofection cells for 3–4 days to let them reach 80%–90% confluency.

***Note:*** Lonza 4D-Nucleofector system can also be used for human iPSC nucleofection. We tested Lonza 4D-nucleofector with P3 Primary Cell 4D-Nucleofector X Kit L for WTC11 iPSCs and turned out with successful nucleofection.

### Generating tagged clonal cell lines with single-cell sorting

**Timing: 4 h (for steps 37 to 41)**

The purpose of this step is to sort the bi-allelically tagged single cell into 96-well plate by Fluorescence-activated Cell Sorting (FACS) and establish the bi-allelically tagged iPSC clones.

37. Prepare Matrigel coated 96-well plate.
38. Remove the Matrigel when the coating is done and add 150 μL of the mTeSR1 medium (with Rock inhibitor and 1% Penicillin-Streptomycin) into each well of the 96-well plate.

***Note:*** We usually prepare 2 plates for one target gene.

**Figure 4:**
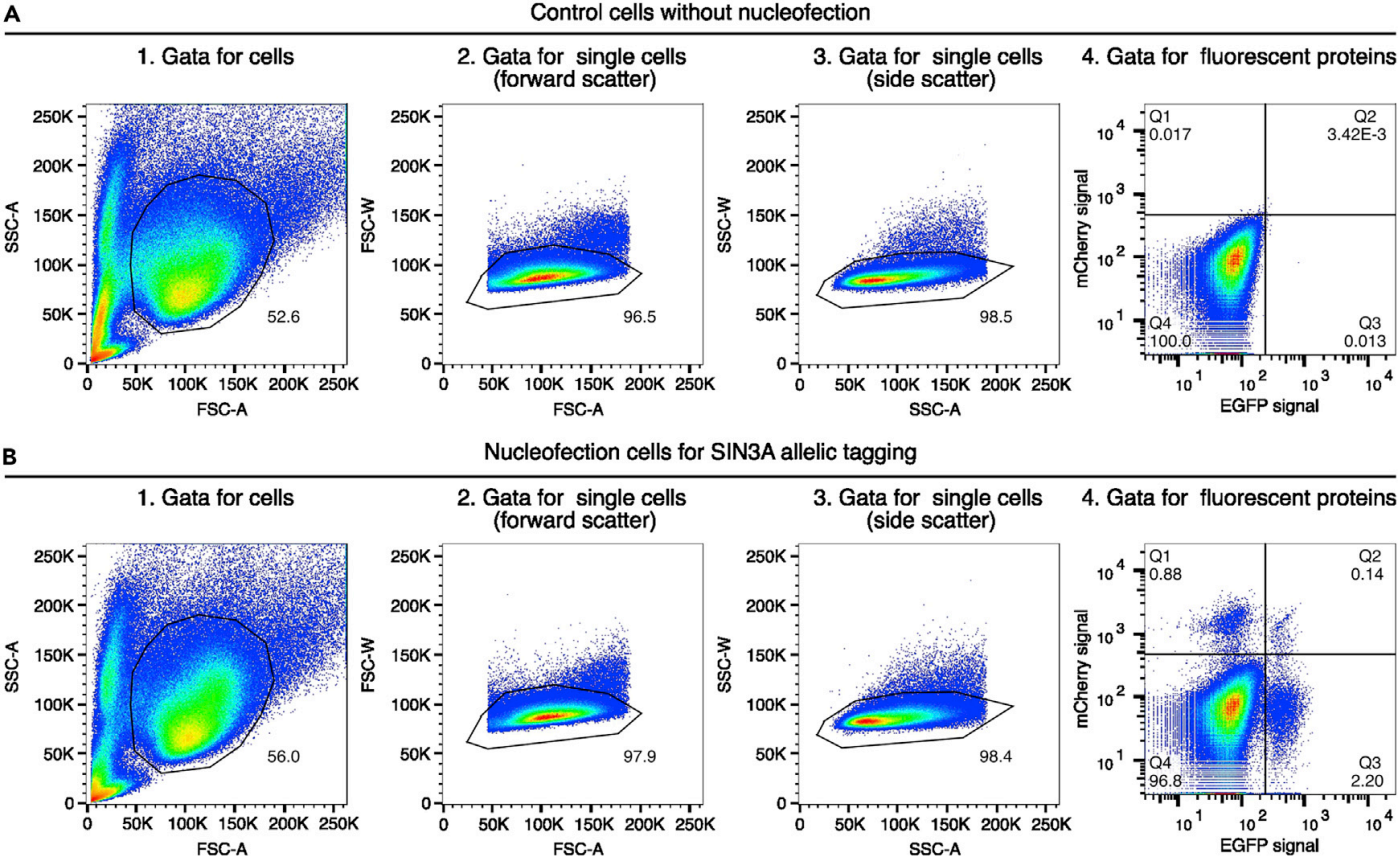
Single-cell sorting strategy of the *SIN3A* bi-allelically tagged cells (A and B) Cells were separated from the debris of various sizes based on the forward scatter area (FSC-A) and side scatter area (SSC-A) (Gata 1: SSC-A versus FSC-A). Then, single cells were separated using a single cell gate based on the width and area metrics of the forward scatter (Gate 2: FSC-W verse FSC-A) and side scatter (Gate 3: SSC-W versus SSC-A). Further, the gata for EGFP and mCherry negative cells were set using control cells without nucleofection (Gate 4: EGFP versus mCherry). Four cell populations were observed in the *SIN3A* nucleofection cell pool compared to the control cells. The EGFP and mCherry double positive population contains the cells with allelic tagging for two alleles of *SIN3A*.

***Note:*** For successful bi-allelic tagging, you should see four cell populations in FACS plot, two single positive populations, one double positive population and one double negative population (Figure 4B). The bi-allelic tagging efficiency may vary among different targets. The percentage of the double positive population for *SIN3A* bi-allelic tagging is about 0.15%.

***Note:*** Plate alignment is a crucial step for plate-based single cell sorting. We noticed the plate sizes vary from different manufacturers. To ensure cells are appropriately collected at the center of each well, please check the plate position by releasing testing droplets to the wells.

39. Detach the nucleofection cells and control cells without nucleofection with Accutase. Resuspend the cells with FACS buffer to 2 × 10^6^ cells/mL.
40. Sort the bi-allelically tagged single cells into the prepared 96-well plate by using BD FACSAria II cell sorter. Please ensure the cell sorter has lasers to detect the fluorescent proteins used for tagging, for example, 488 nm laser for detecting EGFP, 561 nm laser for detecting mCherry (Figure 4).
  a. Use the control cells and set the gate to separate the cells from debris based on the forward scatter area (FSC-A) and side scatter area (SSC-A).
  b. Use the control cells and set the gate for single cells using forward scatter (FSC) and side scatter (SSC).
  c. Set the baseline for EGFP and mCherry signal using control cells.
  d. Switch to the nucleofection cells and set the gate for EGFP and mCherry double positive cells.
  e. Sort the EGFP and mCherry double positive cells from nucleofection cells into 96-well plate with 1 cell/well using single cell mode.

41. Incubate 96-well plates in a 37°C / 5% CO_2_ incubator.

***Note:*** Refresh half media with 1% Penicillin-Streptomycin every 3 days for the first week with a 200 μL multichannel pipette without perturbing the cells. The Penicillin-Streptomycin will inhibit any contamination from sorting. Clones become visible after 1 week. Check the clones with a microscope and mark the clones with only one colony in the well. After one week, refresh half or whole media every 2 days for another week.

### Validating tagged cell lines by genotyping PCR

**Timing: 1 day (for steps 42 to 52)**

The purpose of this step is to verify the bi-allelically tagged clones with genotyping PCR.

42. Design the genotyping primers to confirm the successful bi-allelic tagging.

**Figure 5:**
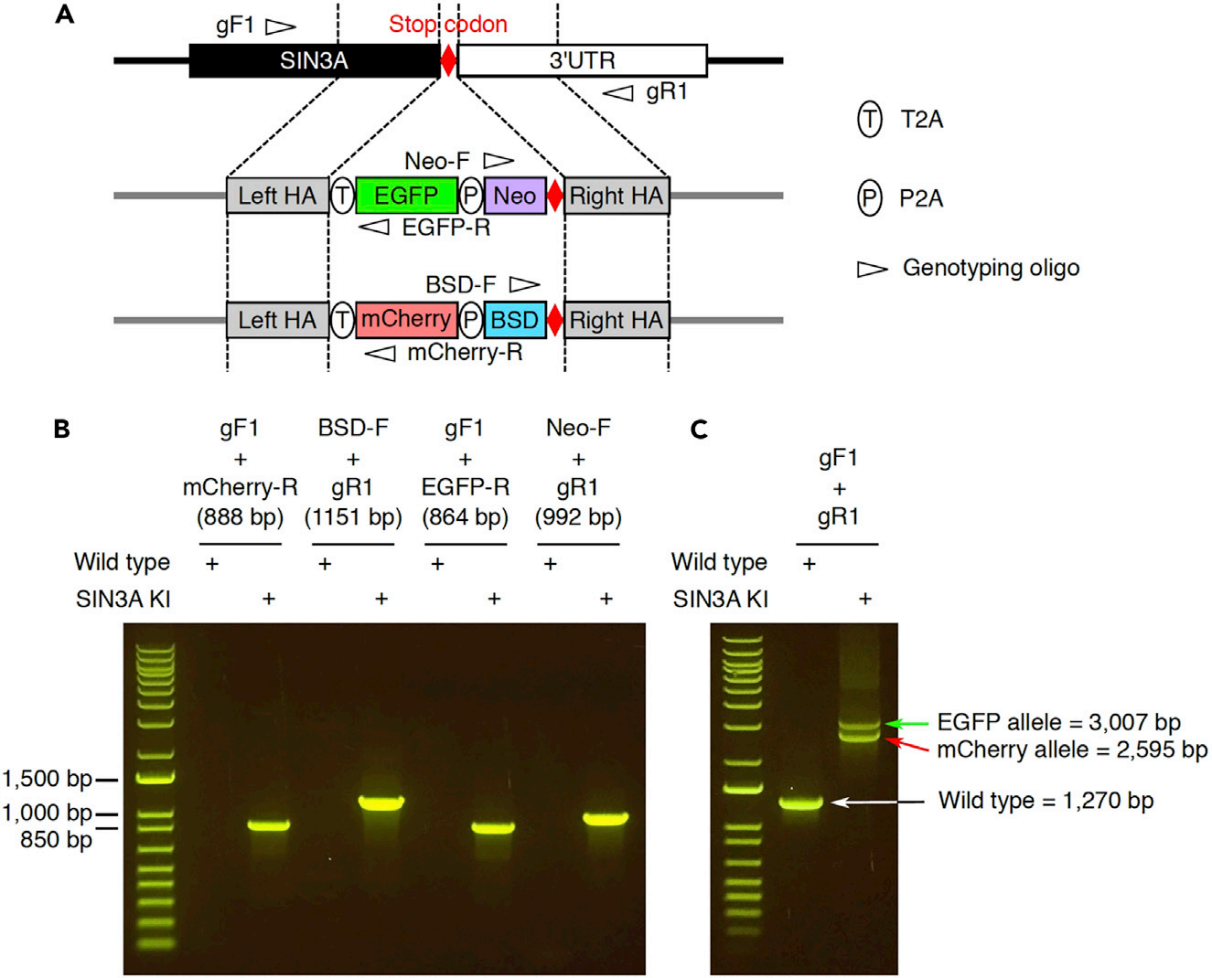
Validation of *SIN3A* bi-allelically tagged clone with genotyping PCR (A) Schematic of the primers designed for genotyping. (B) PCR results demonstrating the successful knock-in of EGFP and mCherry reporters into the *SIN3A* locus. (C) PCR results demonstrating the successful bi-allelic tagging of *SIN3A*.

***Note:*** We designed 4 pairs of genotyping primers for *SIN3A* bi-allelic tagging (Figure 5A, Table 3). For each pair, one primer should target the genomic sequence outside of the homology arm, and another primer should target the insertion sequence, for example, the EGFP, mCherry, neomycin, and blasticidin sequences for *SIN3A* bi-allelic tagging.

43. Start the experimental validation of the clones when they reach about 80% confluency (usually takes 2 weeks). Check the labeled clones in step 41 with a microscope and select the clones with normal morphology for genotyping.

***Note:*** We usually pick 24 clones at one time.

44. Prepare Matrigel coated 24-well plates and add 0.5 mL mTeSR1 medium with Rock inhibitor into each well.
45. Directly scratch the marked wells in a vertical, horizontal, and circular way using a P200 pipet to detach the cells in the well.
46. Pipet up and down gently to mix the cell suspension, then transfer half to the prepared 24-well plate for passaging, and half to the PCR tube for genomic DNA extraction.
47. Extract the genomic DNA.
  a. Spin down the PCR tube with a mini centrifuge to get the cell pellet.
  b. Remove the supernatant and resuspend the cell pellet with 50 μL QuickExtract DNA Extraction Solution.
  c. Incubate the cell suspension at 65°C for 15 min, 98°C for 5 min, and 4°C forever. Then, the lysis can be directly used as PCR template.
48. Set up the genotyping PCR reaction as below.

**Table:** 

Component	Amount (20 μL reaction)
20 μM forward primer	0.5 μL
20 μM reverse primer	0.5 μL
Genomic DNA lysis	5 μL
GoTaq Green Master Mix	10 μL
Nuclease-free water	4 μL

49. Perform amplification using the cycling parameters below.

**Table:** 

Steps	Temperature	Time	Cycles
Initial Denaturation	95°C	2 min	1
Denaturation	95°C	30 s	32
Annealing	55°C	30 s	
Extension	72°C	1 min	
Final extension	72°C	5 min	
Hold	4°C	Forever

50. Check the PCR product with agarose gel electrophoresis.

***Note:*** We checked the *SIN3A* bi-allelic tagging clone with two types of PCR, either amplify the left part and right part of the insertion (Figure 5B), or amplify the whole insertion region (Figure 5C). The amplification of the left part and right part of the insertion confirms the insertion happened in correct genomic location. The amplification of the whole insertion region confirms the whole insertion happed in a bi-allelic manner.

51. Check the DNA sequence of the PCR product with Sanger sequencing (Figure 6).

**Figure 6:**
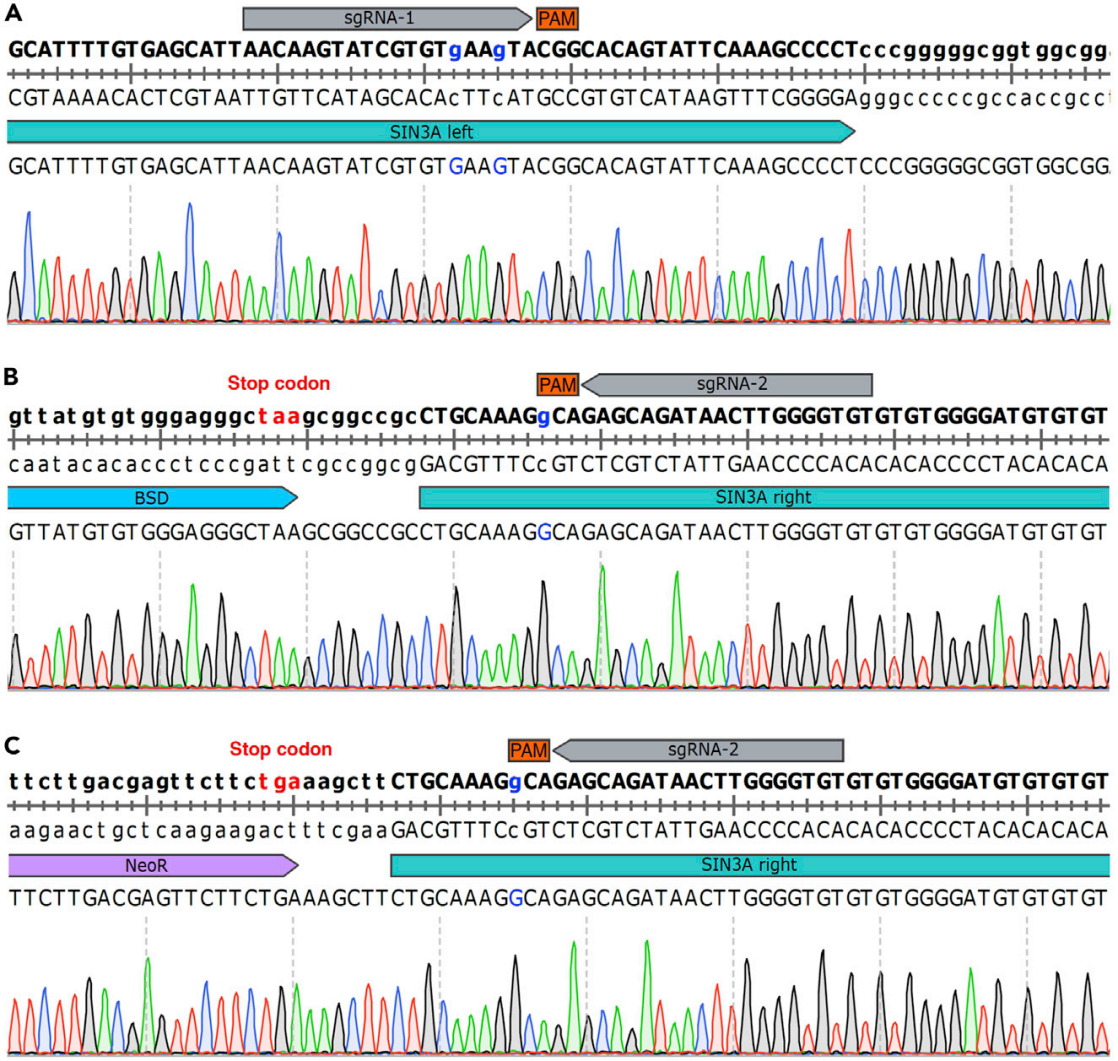
Sanger sequencing results of the genotyping PCR product for the *SIN3A* bi-allelically tagged clone (A–C) Results displaying the successful insertion of designed sequences into the *SIN3A* locus.
52. Expand the correct clones based on the genotyping PCR and Sanger sequencing, and make cryogenic stock for them.

**Table 3:** Oligos for genotyping PCR of *SIN3A* bi-allelically tagged clone

Oligo name	Sequence
gF1	TCCCTCGGTTATACTAACGCATG
gR1	AGCTGGTGGATGGCACTAAG
EGFP-R	CTTCATGTGGTCGGGGTAGC
mCherry-R	CAAGTAGTCGGGGATGTCGG
Neo-F	CGATGATCTCGTCGTGACCC
BSD-F	GTACCGCCACCATGGCCAAGCCTTTGTCTCA

### Functional characterization of the tagged cell lines

**Timing: 2 weeks (for steps 53 to 55)**

The purpose of this step is to characterize the bi-allelically tagged clones using different functional assays. Here, we checked the expression of fluorescent reporters in iPSCs, iPSCs-derived glutamatergic neurons and iPSCs with different passages to confirm the genetic stability of the reporters. We also checked the expression of pluripotency markers in the tagged clones to verify that the reporters do not change the pluripotency of iPSCs.

53. Check the expression of fluorescent reporters from the bi-allelically tagged clones by flow cytometry.
  a. Collect the bi-allelically tagged clones and control cells with Accutase and resuspend the cells with FACS buffer to 2 × 10^6^ cells/mL.
  b. Perform flow cytometry analysis for the clones with BD LSRFortessa cell analyzer, or other flow cytometry cell analyzer. Set the single cell gate and baseline of each fluorescent protein by using the control cells same as step 40.

**Figure 7:**
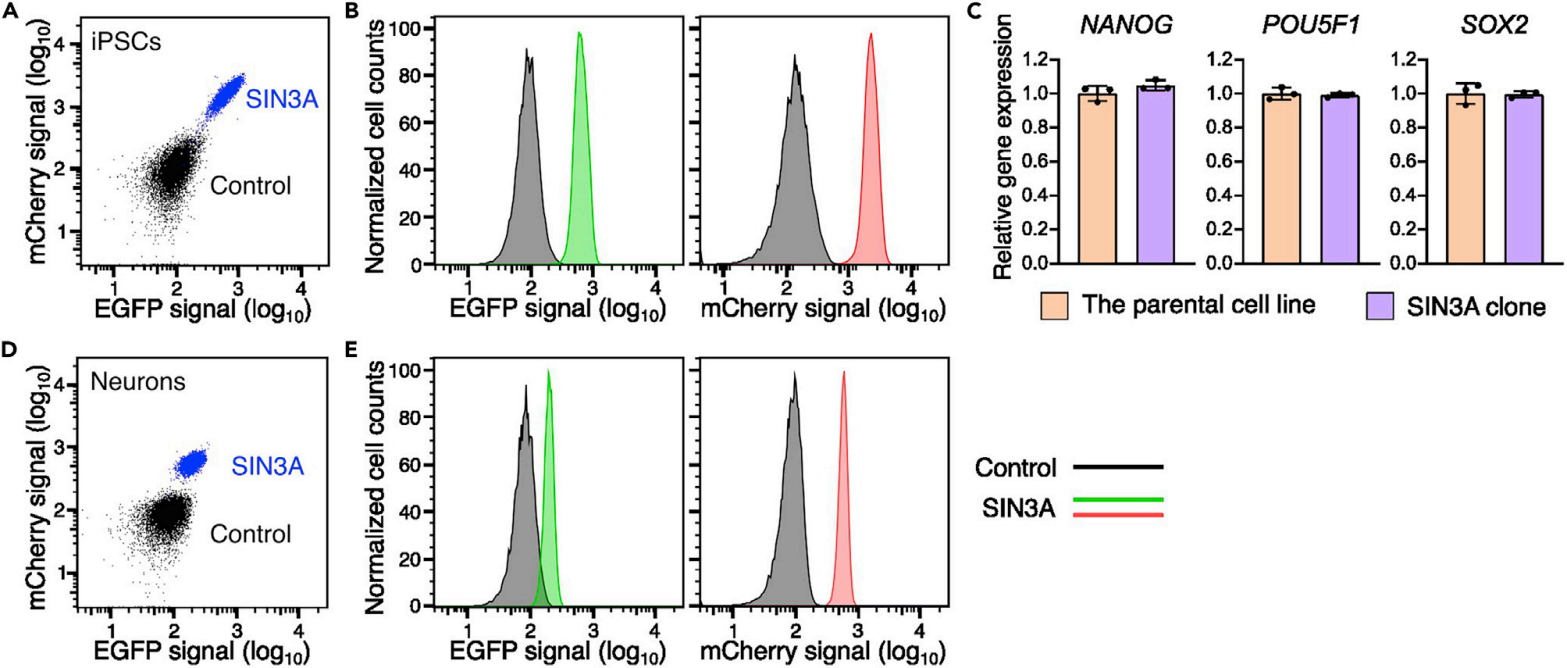
Functional characterization of the *SIN3A* tagged cell line (A and B) Flow cytometry analysis of the *SIN3A* bi-allelically tagged clone. The iPSCs of SIN3A clone show positive EGFP and mCherry signals in the 2D plot (A) and histogram (B) of the flow cytometry analysis. (C) RT-qPCR results show the expression levels of *NANOG*, *POU5F1*, and *SOX2* are the same between the *SIN3A* bi-allelically tagged clone and the parental cell line. Error bars represent the s.d. (D and E) Flow cytometry analysis of the differentiated neurons from *SIN3A* bi-allelically tagged clone. The neurons show positive EGFP signal and mCherry signals in the 2D plot (D) and histogram (E) of the flow cytometry analysis.

***Note:*** For *SIN3A* bi-allelically tagged clone, we saw both EGFP signal and mCherry signal, which indicate the successful expression of fluorescent reporters from *SIN3A* locus (Figures 7A and 7B).

54. Check the expression of pluripotency markers in the bi-allelically tagged clones.
  a. Extract the total RNA from the bi-allelically tagged clone using the QIAGEN RNeasy Plus Mini Kit following the manufacturer’s protocol (https://www.qiagen.com/us/Resources/ResourceDetail?id=1d882bbe-c71d-4fec-bdd2-bc855d3a4b55&lang=en).
  b. Perform reverse-transcription reaction to make cDNA from the extracted total RNA using the iScript cDNA synthesis kit following the manufacturer’s protocol (https://www.bio-rad.com/webroot/web/pdf/lsr/literature/4106228.pdf).
  c. Run qPCR to check the expression of the pluripotency markers from both control cell and tagged clone.

***Note:*** We checked the expression of *NANOG*, *POU5F1* and *SOX2* in the parental cell line and the bi-allelically tagged clone and did not observe a significant difference, which indicates the insertion of fluorescent reporters do not influence the pluripotency of the tagged clone (Figure 7C).

55. Check the genetic stability of the bi-allelically tagged clones during cell culture cycle and differentiation.
  a. Thaw the clone from the cryogenic stock made in step 52 by following the step 5 in before you begin part.
  b. Passage the clone 3 times and check the expression of fluorescent reporters by flow cytometry to make sure the tagged fluorescence reporters can be stably maintained during cell culture cycle.
  c. Check the genetic stability of the bi-allelically tagged clones during differentiation. The detailed protocol for differentiation is available from ENCODE portal (https://www.encodeproject.org/documents/d74fb151-366c-4450-9fa0-31cc614035f9/).
  d. Seed 2 × 10^6^ cells of the bi-allelically tagged clone in one Matrigel-coated well in 6-well plate, and feed the cells with Pre-differentiation Medium for three days.
  e. Dissociate the pre-differentiated cells using Accutase. Seed the dissociated cells into Poly-L-Ornithine-coated plates with 1 × 10^6^ cells per well in 6-well plate. Maintain the differentiation culture for two weeks.
  f. Perform flow cytometry analysis for the differentiated glutamatergic neurons with BD LSRFortessa cell analyzer, or other flow cytometry cell analyzer. Set the single cell gate and baseline of each fluorescent proteins by using the control cells same as step 40.

***Note:*** For the differentiated glutamatergic neurons from *SIN3A* bi-allelically tagged clone, we saw both EGFP signal and mCherry signal, which indicate the fluorescent reporters can be maintained during neuronal differentiation (Figures 7D and 7E).

## Expected outcomes

Tagging endogenous genes is a powerful technology for monitoring gene expression and characterizing protein function. The bi-allelic tagging enables real-time monitoring gene expression and protein subcellular localization in an allelic manner, characterizing *cis* vs. *trans* elements that affect the gene expression, and facilitate pooled CRISPR screening to identified genetic components essential for phenotypes associated with target gene expression. The expected outcome of this protocol will be the bi-allelically tagged clones for the gene-of-interest. Tagging the target gene with one fluorescent protein results in both homozygous and heterozygous knock-in. The cells with heterozygous knock-in could have undesired indel on the non-tagged allele. Two alleles tagging can minimize such unwanted editing events. Furthermore, this protocol can be applied to simultaneously tag two genes.

We usually get about 0.1% positive rate of double positive cells in human iPSC 3–4 days after transfection without any selection. Compared to double positive cells, the positive rate of cells with only one fluorescent protein is about 6 to 16-fold higher. For any batch of transfection, we only need 100,000 cells to sort a full plate of single cells into a 96-well plate. From a 96-well plate, we usually get about 30 survival clones and 80% of the established clones have successful knock-in of both EGFP and mCherry fluorescent reporters, making entire tagging strategy highly efficient.

## Limitations

The gene expression level varies among different genes and diverse cell types. We select the positive allelic tagging cells based on the expression of fluorescent proteins driven by the promoter of the gene-of-interest. This strategy is not suitable for silent genes. To solve this problem, an additional promoter can be added upstream of fluorescent proteins and flanked by loxP sites. The additional promoter can be removed by Cre-mediated excision in the final clones. Additional optimization includes using efficient sgRNA, increasing the transfection efficiency, and antibiotic selection will further increase the success rate.

## Troubleshooting

### Problem 1

Rampant cell death after nucleofection (section “deliver CRISPR-Cas9 genome editing reagents into cells using nucleofection”, steps 35, 36).

### Potential solution

Double check the nucleofection program is optimized for your cell line. Double check the quality of donor vectors by gel electrophoresis and NanoDrop. Purify the donor vectors again if the quality is not good for nucleofection.

### Problem 2

The bi-allelic tagging efficiency is low (section “generating tagged clonal cell lines with single cell sorting”, step 40).

### Potential solution

Grow the nucleofection cells longer to get more cells for sorting. Treat the nucleofection cells with antibiotics when antibiotic resistance genes are added in the donor vector to enrich the cells with successful tagging. Use longer homology arms to increase tagging efficiency. Test sgRNA cutting efficiency *in vitro* and select the effective sgRNA for nucleofection. Reduce the size of the donor vectors to increase the transfection efficiency of donor vectors. Test the transfection efficiency using a control plasmid, for example the pmaxGFP vector in LONZA human stem cell nucleofector kit.

### Problem 3

The low survival rate from single cell sorting in 96-well plate (section “generating tagged clonal cell lines with single cell sorting”, step 41).

### Potential solution

Sort single cells into multiple 96-well plates to get more survival clones. Add live cell marker in cell suspension for sorting, for example, SYTOX Blue or DAPI, and exclude the dead cells based on the signal of the live cell marker when setting up the gate for single cell sorting. Use the media specifically designed for improving the survival of human iPSCs in single-cell workflows, for example, CloneR, CloneR 2.

### Problem 4

No band or only non-specific band is amplified (section “validating tagged cell lines by genotyping PCR”, step 50).

### Potential solution

Optimize the PCR program or use another DNA polymerase. Purify or clean the extracted genomic DNA to provide the high quality DNA template for PCR. Design and order more oligos for PCR.

### Problem 5

The Sanger sequencing reaction is failed or shows mixed peaks (section “validating tagged cell lines by genotyping PCR”, step 51).

### Potential solution

Amplify more PCR product and clear the PCR product using gel extraction. Extract the genomic DNA using other genomic DNA traction kit. Make sure the cell population is clonal.

## Resource availability

### Lead contact

Further information and requests for resources and reagents should be directed to and will be fulfilled by the lead contact, Yin Shen (yin.shen@ucsf.edu).

### Materials availability

The EGFP and mCherry donor backbone used in this study are available on request.

## Data Availability

This study did not generate or analyze any datasets or code.
